# Thiamine Acquisition Strategies Impact Metabolism and Competition in the Gut Microbe *Bacteroides thetaiotaomicron*

**DOI:** 10.1128/mSystems.00116-17

**Published:** 2017-09-26

**Authors:** Zachary A. Costliow, Patrick H. Degnan

**Affiliations:** Department of Microbiology, University of Illinois at Urbana-Champaign, Urbana, Illinois, USA; University of Chicago

**Keywords:** competition, microbiome, *pnuT*, thiamine pyrophosphate (TPP), vitamin B_1_

## Abstract

Thiamine (vitamin B_1_) is an essential cofactor for all organisms. Humans primarily acquire thiamine through their diet, and thiamine deficiencies have adverse neurological effects. However, the role gut microbes play in modulating thiamine availability is poorly understood, and little is known about how thiamine impacts the stability of microbial gut communities. To investigate thiamine’s role in the gut, we utilized the model gut microbe *Bacteroides thetaiotaomicron*. Transcriptome sequencing (RNA-seq) revealed a global downregulation of thiamine and amino acid biosynthesis, glycolysis, and purine metabolism when thiamine was present. Using genetic mutants with thiamine biosynthesis and transport locus mutations, we determined both systems were critical for growth in thiamine-deficient medium. The defect in the double transport mutant suggests an uncharacterized feedback mechanism between thiamine transport and biosynthesis in *B. thetaiotaomicron*. Mutant phenotypes were recapitulated during pairwise competitions, reinforcing the importance of encoding versatile thiamine acquisition mechanisms when thiamine concentrations are variable. In addition, liquid chromatography-mass spectrometry (LC-MS) analyses corroborate that exogenous thiamine levels affect the internal thiamine pool of *B. thetaiotaomicron*. Furthermore, we computationally examined the ability of other gut microbes to acquire thiamine and identified lineage-specific differences in thiamine acquisition strategies. Among the *Bacteroidetes*, the capacities for both thiamine transport and biosynthesis are common. Together, these data show that thiamine acquisition mechanisms used by *B. thetaiotaomicron* not only are critical for its physiology and fitness but also provide the opportunity to model how other gut microbes may respond to the shifting availability of thiamine in the gut.

**IMPORTANCE** Variation in the ability of gut microbes to transport, synthesize, and compete for vitamin B_1_ (thiamine) is expected to impact the structure and stability of the microbiota, and ultimately this variation may have both direct and indirect effects on human health. Our study identifies the diverse strategies employed by gut *Bacteroidetes* to acquire thiamine. We demonstrate how the presence or absence of thiamine biosynthesis or transport dramatically affects the abundance of *B. thetaiotaomicron* in a competitive environment. This study adds further evidence that altering the presence or concentrations of water-soluble vitamins such as thiamine may be an effective method for manipulating gut community composition. In turn, targeted thiamine delivery could be used therapeutically to alter dysbiotic communities linked to disease.

**Author Video**: An author video summary of this article is available.

## Abstract (precis)

Variation in the ability of gut microbes to transport, synthesize, and compete for vitamin B_1_ (thiamine) is expected to impact the structure and stability of the microbiota, and ultimately this variation may have both direct and indirect effects on human health. Our study identifies the diverse strategies employed by gut *Bacteroidetes* to acquire thiamine. We demonstrate how the presence or absence of thiamine biosynthesis or transport dramatically affects the abundance of *B. thetaiotaomicron* in a competitive environment. This study adds further evidence that altering the presence or concentrations of water-soluble vitamins such as thiamine may be an effective method for manipulating gut community composition. In turn, targeted thiamine delivery could be used therapeutically to alter dysbiotic communities linked to disease.

## INTRODUCTION

Thiamine, a small metabolite vitamin is an essential cofactor in all three domains of life. Thiamine is necessary for glycolysis, the tricarboxylic acid (TCA) cycle, branched-chain amino acid metabolism, and nucleotide metabolism (1). Deficiencies of thiamine in humans can lead to neurodegenerative disorders, such as Wernicke’s encephalopathy and beriberi, as well as heart failure, ataxia, and paralysis (2). In humans, thiamine is believed to be primarily derived from dietary sources, such as protein-rich foods as well as fortified grains (2), and is absorbed throughout the small and large intestines (3). However, there is evidence that thiamine availability is modulated by or acquired from the dense and diverse microbial communities present in the gastrointestinal tract (3–5).

Competition for and production of small metabolites such as vitamins, amino acids, and short-chain fatty acids influence the composition (e.g., abundance and types) of microbes that inhabit the gastrointestinal tract (6–8). As such, small metabolites are therapeutic candidates for treatment of diseases associated with radical changes in gut microbial community composition, such as Alzheimer’s, diabetes, and obesity (9–11). Indirect evidence suggests thiamine may influence gut community structure. Metagenomic analysis of human cohorts of different ages determined that not only did overall microbial communities change with age, but the abundance of microbial thiamine biosynthesis genes increased with the age of the host (12). Conversely, in a separate study the activity of human intestinal thiamine transporters was observed to decrease with age (13). These observations may indicate a lower requirement for thiamine by the host with age or possibly an increase in thiamine availability due to microbial production in older individuals. While these correlative data exist, little is known mechanistically about how the competition for and production of thiamine impact individual gut microbes like *Bacteroides thetaiotaomicron*, microbial gastrointestinal communities, or human health.

Microbial thiamine biosynthesis is a bifurcated pathway involving the synthesis of two precursors that are condensed to form thiamine (14). Microbes that lack complete *de novo* biosynthesis of thiamine often encode partial biosynthetic pathways of the precursors thiazole and hydroxymethyl pyrimidine phosphate or simply phosphorylate free thiamine into the biologically active form, thiamine pyrophosphate (TPP) (15). Organisms that lack or have incomplete biosynthetic pathways require transporters for thiamine or its precursors. Humans exclusively transport free thiamine in the proximal small intestine as well as in the large intestine (13), while some microbes like *Salmonella enterica* use an ABC transporter to acquire thiamine and its phosphorylated forms (16). However, despite our understanding of thiamine biosynthesis and metabolism in humans and a limited number of model organisms, little is known about thiamine acquisition strategies among most beneficial gut microbes.

Initial comparative genomic analyses of several gut microbes suggested that the *Bacteroidetes*, a phylum that can comprise as much as 50% of the gastrointestinal community (17), possess a canonical thiamine biosynthesis pathway (18). In addition, a novel transport system was predicted due to the presence of *cis*-acting RNA regulatory elements, TPP riboswitches, preceding putative transport genes (18). However, limited investigation of thiamine acquisition in the *Bacteroidetes* has been attempted (19). Here, we have carried out a bioinformatic analysis of 641 gut-associated microbes and performed a transcriptomic, genetic, and competitive characterization of the prominent gut microbe *B. thetaiotaomicron* in response to thiamine. In turn, we have characterized (i) the effect of thiamine on the transcriptome, (ii) the predicted thiamine biosynthesis and transport genes, (iii) the competitive defects of biosynthesis and transport mutants, and (iv) the effects that exogenous thiamine has on the internal thiamine pool of *B. thetaiotaomicron*.

## RESULTS

### Global gene expression response of *B. thetaiotaomicron* to thiamine availability.

**FIG 1:**
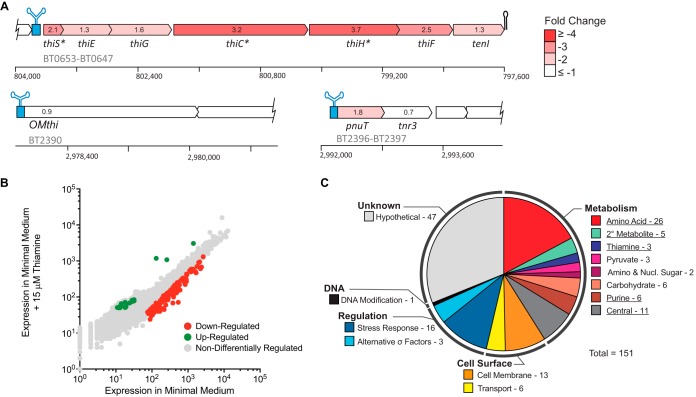
Transcriptomic response of *B. thetaiotaomicron* to exogenous thiamine. (A) A schematic of TPP riboswitch-regulated operons is shown with expression fold changes indicated and colored according to the heat map. Asterisks indicate individual genes that are significantly downregulated (*q* ≤ 0.05) in the presence of thiamine. (B) Genome-wide expression response of *B. thetaiotaomicron* to thiamine. Gray circles indicate genes that are not significantly up- or downregulated, red circles are genes significantly downregulated in the presence of thiamine, and green circles are genes significantly upregulated (*q* ≤ 0.05). (C) KEGG gene clusters that contain genes which are significantly downregulated. The numbers to the right of each KEGG pathway denote the number of genes downregulated in each category. Underlined categories indicate KEGG pathways that are significantly downregulated through GSEA analysis (*P* ≤ 0.05).

*B. thetaiotaomicron*, a prominent gut microbe, is typical of the *Bacteroidetes* in its thiamine acquisition strategy, containing TPP riboswitch-regulated biosynthesis (*thiSEGCHF-tenI* [*BT0653* to *BT0647*]), putative outer membrane transporter (*OMthi* [*BT2390*]), and putative inner membrane transporter, and thiamine pyrophosphorylase (*pnuT-tnr3* [*BT2396* to *BT2397*]) operons (18). TPP riboswitches can act at the transcriptional or translational level (20), and it is uncertain how they function in the *Bacteroidetes*. To investigate the response of *B. thetaiotaomicron* to the availability of thiamine, strand-specific transcriptome sequencing (RNA-seq) was performed on cultures grown in minimal medium supplemented with either 0 or 15 µM thiamine in duplicate. A total of 34,890,999 reads were recovered, ranging from approximately 8 million to 9.5 million reads per sample that were quality filtered, trimmed, and analyzed with Rockhopper (see Table S1 in the supplemental material) (21). Of the 4,778 coding sequences in the *B. thetaiotaomicron* genome, 151 showed a ≥2-fold expression change (false-discovery rate [FDR], *q* ≤ 0.05). Of the three putative TPP riboswitch-regulated operons, the biosynthetic operon as a whole was significantly downregulated when thiamine was present (Fig. 1A). No significant differential expression was observed at either of the predicted transport operons (Fig. 1A). This is in striking contrast to the multiple vitamin B_12_ riboswitch-regulated B_12_ transporters in *B. thetaiotaomicron*, which exhibit a strong transcriptional induction when grown under vitamin B_12_-limiting conditions (6). However, this does not rule out their involvement in thiamine transport, particularly as the putative inner membrane transporter, PnuT, has been heterologously expressed in *Escherichia coli* and demonstrated to transport thiamine (19).

10.1128/mSystems.00116-17.6 TABLE S1  Samples used for the RNA sequencing experiment. Download TABLE S1, XLS file, 0.02 MB. Copyright © 2017 Costliow and Degnan. 2017 Costliow and Degnan This content is distributed under the terms of the Creative Commons Attribution 4.0 International license.

In addition to the genes in the biosynthetic operon, 148 other genes were differentially expressed due to exogenous thiamine in the environment. In fact, 132 of the 151 differentially regulated genes were downregulated when 15 µM thiamine was present in the medium (Fig. 1B). Using gene set enrichment analysis (GSEA), we determined if specific pathways are differentially expressed due to thiamine availability (22). Significantly downregulated KEGG pathways in the presence of exogenous thiamine were observed in thiamine metabolism, amino acid biosynthesis, purine metabolism, the TCA cycle, coenzyme A biosynthesis, and pyruvate metabolism (FDR-corrected *P* ≤ 0.001) (Fig. 1C). This suggests that in the presence of excess exogenous thiamine, *B. thetaiotaomicron* downregulates transcription of the thiamine biosynthetic pathway in order to maintain homeostatic levels of internal thiamine. In addition to this, *B. thetaiotaomicron* also decreases transcription of thiamine-dependent pathways such as branched-chain amino acid biosynthesis, glycolysis, and the citric acid cycle when excess thiamine is present. This decreased expression in the presence of excess thiamine is counterintuitive to what was expected. Normally, it is expected that expression of cofactor-dependent enzymes is positively correlated with the concentration of the cofactor (e.g., iron) (23). In the case of thiamine in *B. thetaiotaomicron*, we observe the opposite. The expression of thiamine-dependent enzymes was inversely correlated.

### Genetic analysis of thiamine acquisition genes in *B. thetaiotaomicron.*

To assess the conditions under which thiamine biosynthesis and uptake are important for the growth of *B. thetaiotaomicron* and validate the role of the predicted transporters in thiamine uptake, a series of in-frame genetic deletions were generated. We focused on the three TPP riboswitch-regulated operons, removing the entirety of the downstream biosynthetic or putative transport coding sequences. The resulting mutant strains and their complements (Table 1) were evaluated for their ability to grow under a range of exogenous thiamine concentrations.

**TABLE 1:** Deletion loci and complements generated in this study

Strain type	Description
WT	Wild-type deletion background, Δ*BT2275* FUdR^r^, intact thiamine biosynthetic and transport operons^a^
Deletion mutants	
ΔBioThi	Deletion of thiamine biosynthetic operon *thiSEGCHF-tenI* (*BT0647–BT0653* [797661–803616])
Δ*OMthi*	Deletion of putative outer membrane, TonB-dependent thiamine transporter *BT2390* (2977834–2980058)
Δ*pnuT*	Deletion of putative inner membrane thiamine transporter *BT2396* (2992218–2992778)
ΔΔBioThi-*OMthi*	Deletion of *BT0647–BT0653* and *BT2390*
ΔΔBioThi-*pnuT*	Deletion of *BT0647–BT0653* and *BT2396*
ΔΔTransport	Deletion of *BT2390* and *BT2396*
ΔΔΔ	Deletion of *BT0647–BT0653*, *BT2390*, and *BT2396*
Complementation constructs	
ΔBioThi+pBioThi	*BT0647–BT0653* complemented in *trans*
ΔΔBioThi-*OMthi*+pBioThi	*BT0647–BT0653* complemented in *trans*
ΔΔBioThi-*pnuT*+pBioThi	*BT0647–BT0653* complemented in *trans*
ΔΔTransport+p*OMthi*	*BT2390* complemented in *trans*
ΔΔTransport+p*pnuT*	*BT2396* complemented in *trans*
ΔΔTransport+p*pnuT*+*OMthi*	*BT2396* and *BT2390* complemented *in trans*

a For details, see reference 33.

**FIG 2:**
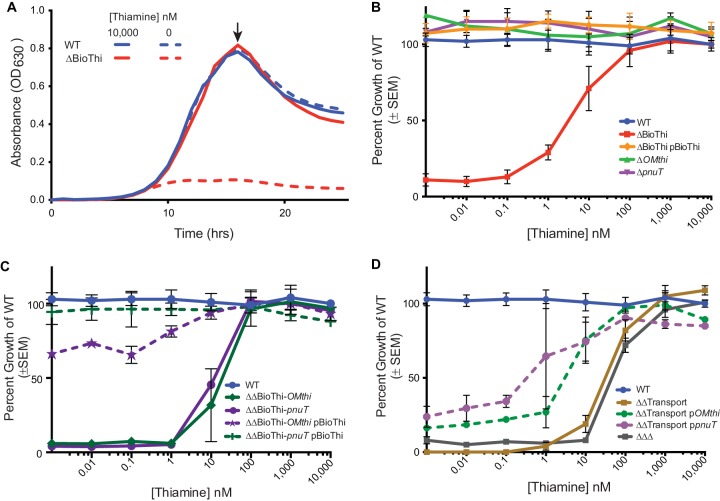
Growth phenotypes of *B. thetaiotaomicron* thiamine acquisition mutants. Thiamine acquisition operons have differential effects on the ability of *B. thetaiotaomicron* to grow under thiamine-limiting conditions. (A) Representative growth curve of the wild-type (WT) and ΔBioThi strains showing growth defects of the ΔBioThi mutant in a thiamine-deficient environment (0 nM) and a thiamine-replete environment (10,000 nM). An arrow indicates the maximal growth point at ~17.5 h used to calculate the percentage of growth of the WT in minimal medium supplemented with 10,000 nM thiamine to compare growth phenotypes across concentrations of thiamine in panels B to D. (B) Single mutants with mutation of the outer membrane transporter (Δ*OMthi*) and inner membrane transporter (Δ*pnuT*) loci showed no defect, while ΔBioThi strains were variably attenuated in thiamine concentrations of <100 nM, and complementation of ΔBioThi with the entire loci with the native promoter expressed in *trans* can fully complement the mutant. (C) Mutants lacking biosynthesis (ΔBioThi) and a single transport gene (*pnuT* or *OMthi*) have severe growth phenotypes at <100 nM thiamine but can be complemented with the biosynthesis locus under its native promoter to wild-type or near-wild-type levels. (D) The double transport mutant (ΔΔTransport) and triple locus mutant (ΔΔΔ) were both highly attenuated in thiamine concentrations of <1,000 nM. Complementation of single transporters in the ΔΔTransport background can partially rescue the severe growth phenotype. SEM, standard error of the mean.

Wild-type *B. thetaiotaomicron* readily grows in media without added thiamine (Fig. 2A) (24), which corresponds with elevated expression of the TPP riboswitch-regulated *thiSEGCHF-tenI* operon identified by RNA-seq. Therefore, deletion of the major thiamine biosynthetic operon (ΔBioThi [*BT0653* to *BT0647*]) is expected to completely inhibit growth in minimal medium without thiamine. Indeed, growth inhibition occurs in the ΔBioThi mutant, severely attenuating terminal cell densities (Fig. 2A and B). Partial growth of the ΔBioThi mutant is recovered when the medium is supplemented with ≥1 nM thiamine (Fig. 2B). The calculated 50% effective concentration (EC_50_: the effective concentration of thiamine at which the terminal optical density at 600 nm [OD_600_] of the mutant attains 50% of the wild-type level) (see Table S2 in the supplemental material) for the ΔBioThi mutant is 2.8 nM (95% confidence interval [CI], 1.4 to 5.5 nM), showing that biosynthesis is critical in thiamine-deficient environments. This growth defect can be complemented when the *thiSEGCHF-tenI* operon and its native promoter are returned to the ΔBioThi mutant in *trans* (Fig. 2B), alleviating the requirement for exogenous thiamine and returning the EC_50_ to 0 nM thiamine (Table S2). Together these data suggest that *B. thetaiotaomicron* does in fact encode thiamine transporters that likely function in the low-nanomolar range. This is comparable to other characterized thiamine transporters such as ThiPQ-TbpA in *Salmonella enterica* serovar Typhimurium (16).

10.1128/mSystems.00116-17.7 TABLE S2  Effective concentrations for 50% WT growth represented as nanomolar concentration of thiamine required. Download TABLE S2, XLS file, 0.1 MB. Copyright © 2017 Costliow and Degnan. 2017 Costliow and Degnan This content is distributed under the terms of the Creative Commons Attribution 4.0 International license.

To determine if the predicted TPP riboswitch-regulated inner (*pnuT* [*BT2396*]) and outer (*OMthi* [*BT2390*]) membrane transporters are responsible for transporting thiamine, individual transporter deletions were constructed in the wild-type background (Δ*pnuT* and Δ*OMthi*). Regardless of the thiamine concentration in the medium, growth of the single transporter mutants never significantly varied from wild type (Fig. 2B). This result was expected as the thiamine biosynthesis pathway is fully intact. Therefore, to further investigate the role of these transporters, double mutants consisting of either the biosynthetic operon and a single transporter or both transporters were generated.

Deletion of the biosynthetic operon in conjunction with either the inner membrane (ΔΔBioThi-*pnuT*) or outer membrane (ΔΔBioThi-*OMthi*) transporters resulted in mutants that required ~10 times more thiamine (1,000 nM) than the ΔBioThi mutant to achieve growth equal to that of the wild type (Fig. 2C). The EC_50_ for the double mutants increased to 15.8 nM (95% CI, 8.1 to 31 nM) thiamine for the biosynthesis and outer membrane transporter mutant and to 10.9 nM (95% CI, 9.2 to 13 nM) for the biosynthesis and inner membrane transporter double mutant compared to an EC_50_ of 2.8 nM (95% CI, 1.4 to 5.5 nM) in the biosynthesis mutant (Table S2). Thus, partial disruption of thiamine transport in the absence of a complete biosynthetic pathway further attenuates but does not completely abolish thiamine-dependent growth. Growth of either of these two mutant backgrounds can be fully complemented by restoring the major thiamine biosynthesis operon in *trans* (Fig. 2C).

Compared to the individual biosynthesis and transporter mutants, deletion of both transporters while the biosynthesis locus is fully intact (ΔΔTransport) has a highly deleterious effect on growth (Fig. 2D). This mutant requires at least 10 µM thiamine to achieve wild-type growth and has an EC_50_ of 30.3 nM (95% CI, 22.8 to 40.3 nM) (Table S2). To ensure this phenotype was not due to an inability to transport cysteine, the only thiamine precursor present in the minimal medium, the double transport mutant was grown with an alternate sulfur source. The growth phenotype of the mutant in the modified medium was unchanged, suggesting that neither transporter is likely involved in cysteine transport (see Text S1 and Fig. S1 in the supplemental material).

10.1128/mSystems.00116-17.1 TEXT S1  Supplemental results and methods. Download TEXT S1, PDF file, 0.1 MB. Copyright © 2017 Costliow and Degnan. 2017 Costliow and Degnan This content is distributed under the terms of the Creative Commons Attribution 4.0 International license.

10.1128/mSystems.00116-17.2 FIG S1  Putative *B. thetaiotaomicron* thiamine transporters are specific for thiamine. Download FIG S1, PDF file, 0.2 MB. Copyright © 2017 Costliow and Degnan. 2017 Costliow and Degnan This content is distributed under the terms of the Creative Commons Attribution 4.0 International license.

Individual complementation constructs were generated for the inner and outer membrane components and introduced in *trans* into the double transport mutant (ΔΔTransport+p*pnuT* and ΔΔTransport+p*OMthi*). Although we did not observe full complementation by either of the two constructs, they did significantly improve the EC_50_ from 30.3 nM (95% CI, 22.8 to 40.3 nM) in the double transport mutant to 1.9 nM (95% CI, 0.8 to 4.4 nM) and 0.1 nM (95% CI, 0 to 1.9 nM) (Table S2) in the outer and inner membrane transport complements, respectively (*t* test, *P* < 0.0001) (Fig. 2D). In addition to single transport complements, a double transport complement (ΔΔTransport+p*pnuT+OMthi*) was made and tested with similar results to the single transport complements. The double complement allowed for better fitness at low levels of thiamine, achieving >35% of wild-type growth at 0 nM added thiamine and greater than 80% of wild-type growth at 10 nM (see Fig. S2 in the supplemental material). It also significantly improved the ΔΔTransport mutant’s EC_50_ from 30.3 nM to 0.1 nM (95% CI, 0 to 0.2 nM) (Table S2). While not uncommon, it is possible that with introduction of the complementation vectors in *trans*, the complements were not expressed at the same level as the wild type or are missing key upstream elements preventing the strains from growing as well as wild-type *B. thetaiotaomicron* (Fig. 2D).

10.1128/mSystems.00116-17.3 FIG S2  Growth of dually complemented ΔΔTransport mutant. Download FIG S2, PDF file, 0.1 MB. Copyright © 2017 Costliow and Degnan. 2017 Costliow and Degnan This content is distributed under the terms of the Creative Commons Attribution 4.0 International license.

A final triple mutant of all three TPP-regulated loci was generated (ΔΔΔ). This strain demonstrated a similar level of growth inhibition to the double transport mutant (Fig. 2D). It required 10 µM thiamine in the medium to attain 100% wild-type growth (EC_50_ of 51.9 nM; 95% CI, 42.4 to 63.6 nM) (Table S2). It is important to note that growth defects only affected the terminal density of the mutants, and the doubling times remained fairly constant (~2 h). Together these data confirm the crucial role these operons play in producing and acquiring thiamine.

### Factors affecting the phenotype of the double transport mutant.

**FIG 3:**
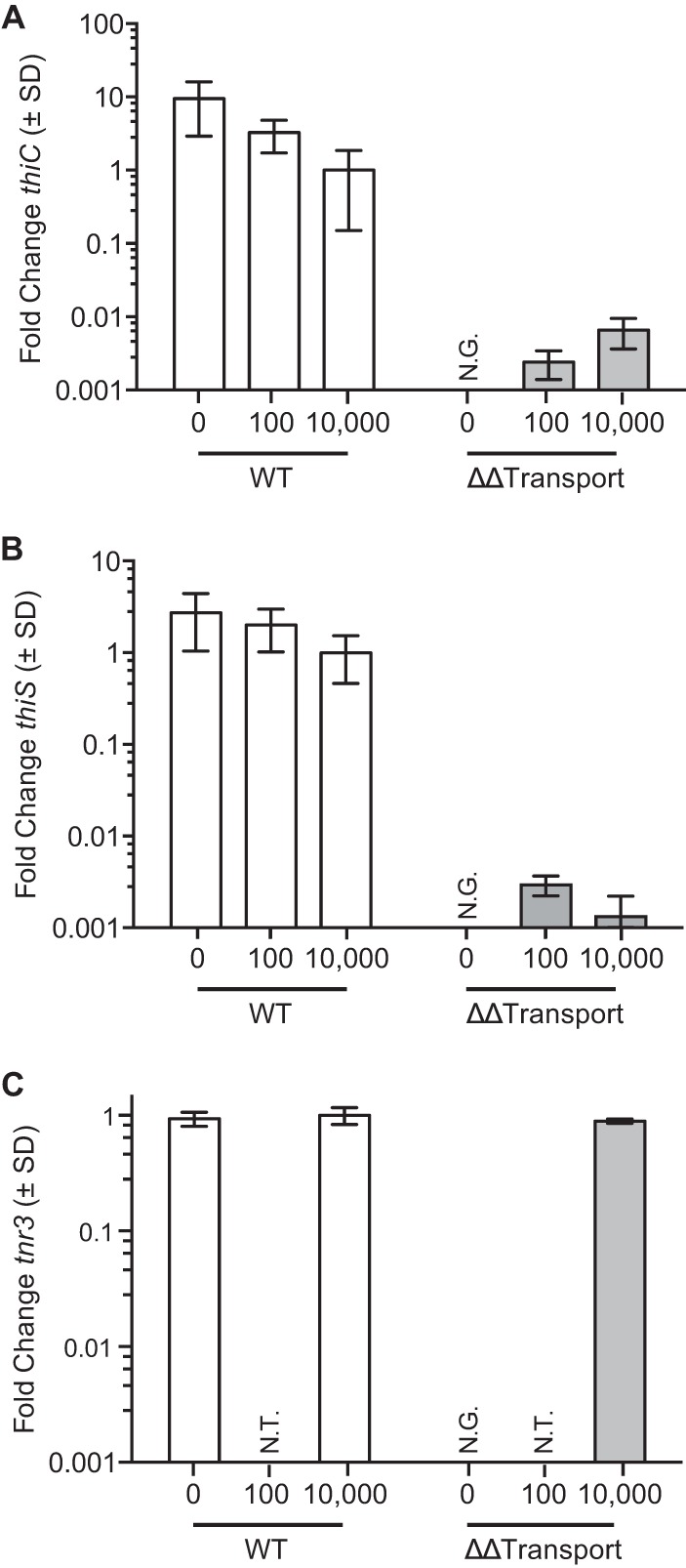
Analysis of thiamine biosynthesis in the wild type and double transport mutant. Expression of biosynthesis was quantitated via RT-qPCR of *thiC* (A) and *thiS* (B) in both wild-type and double transport mutant backgrounds of *B. thetaiotaomicron* at 0, 100, and 10,000 nM concentrations of thiamine for the wild type and 100 and 10,000 nM concentrations of thiamine for the double transport mutant. Expression of *tnr3* was also measured in the wild type and double transport mutant at 0 and 10,000 nM thiamine (C) in order to ensure that the double transport mutant did not have a polar effect on downstream genes. SD, standard deviation; N.G., no growth was achieved to carry out the experiment; N.T., not tested at a certain concentration.

To determine if dysregulation of biosynthesis played a role in the severe growth phenotype of the double transport mutant, reverse transcription-quantitative PCR (RT-qPCR) was performed. Consistent with the RNA-seq data (Fig. 1), expression of the major biosynthetic operon in wild-type *B. thetaiotaomicron* is upregulated under thiamine-deficient conditions (0 and 100 nM) compared to under the replete condition (10,000 nM) (Fig. 3A and B). In a thiamine-deficient environment, gene *thiC* (Fig. 3A) is expressed 9.2-fold higher in 0 nM thiamine than in 10,000 nM and 3.3-fold higher than in 100 nM thiamine (Fig. 3A). Similarly the expression of* thiS* is expressed 2.7-fold to 2-fold higher in 0 nM thiamine than in 10,000 and 100 nM, respectively (Fig. 3B). As noted above the ΔΔTransport mutant cannot be cultured in 0 nM thiamine, but *thiC* and *thiS* expression was determined in 100 and 10,000 nM thiamine. In marked contrast with the wild type, expression of *thiC* and *thiS* is repressed over 100-fold in media with 100 or 10,000 nM thiamine. These data suggest that transport and biosynthesis are linked and likely contribute to the severe growth defect in the ΔΔTransport mutant despite the presence of an intact biosynthetic operon.

In addition, we also performed RT-PCR on *tnr3*, which occurs immediately downstream of *pnuT*, to ensure that the generation of the ΔΔTransport mutant did not have a polar effect (Fig. 3C). The expression of *tnr3* remains unchanged in wild-type and the ΔΔTransport mutant cells regardless of the thiamine concentration tested. Given this result, we infer that the fitness defect is not due to a polar effect of deleting the transport genes.

### Consequences of thiamine acquisition on competition.

**FIG 4:**
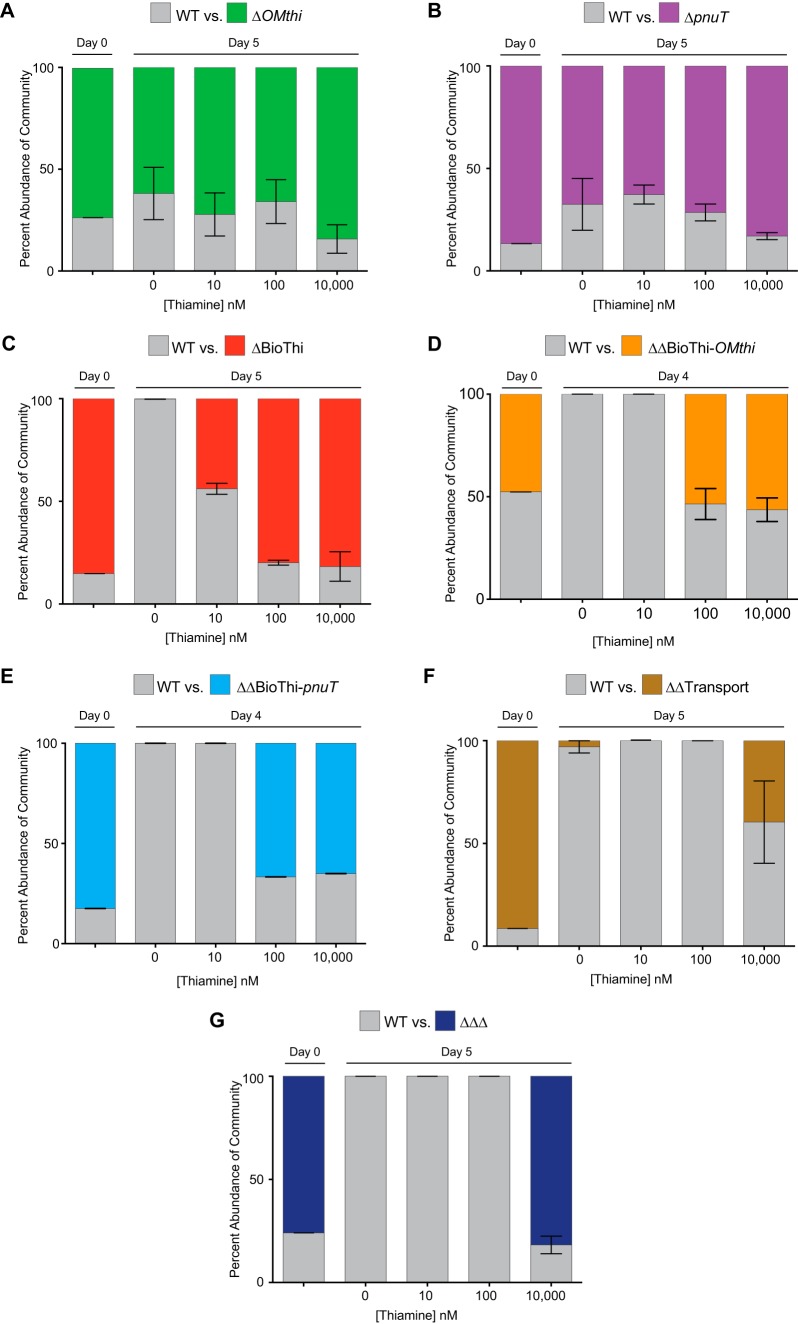
Single competitions between the wild type and thiamine acquisition mutants. Competitions between wild-type *B. thetaiotaomicron* and the (A) Δ*OMthi*, (B) *ΔpnuT*, (C), ΔBioThi, (D) ΔΔBioThi-*OMthi*, (E) ΔΔBioThi-*pnuT*, (E) ΔΔTransport, and (F) ΔΔΔ thiamine acquisition mutants were performed over 4 or 5 days in defined thiamine concentrations. Strain abundances were quantified by qPCR and are represented as a percentage of the entire community at 4 concentrations of thiamine (0, 10, 100, and 10,000 nM). Error bars represent the standard error of the mean from three biological replicates.

We investigated the competitive capacities of the *B. thetaiotaomicron* thiamine acquisition mutants in pairwise competitions with wild-type *B. thetaiotaomicron*. To determine if coculture increased (competition) or decreased (cross-feeding) the individual phenotypes, mixed cultures were serially passaged for 5 days in media with 0, 10, 100, 10,000 nM exogenous thiamine, and strain abundances were monitored through time by qPCR (Fig. 4; see Fig. S3 in the supplemental material). The single transport mutants (Δ*pnuT* and Δ*OMthi*) successfully coexisted with wild-type *B. thetaiotaomicron* under every condition tested (Fig. 4A and B). The mutants maintained 60 to 80% of the population throughout the 5 days of serial passaging. Although the abundance of the mutants fluctuated during passaging, this was likely due to stochastic effects during the daily inoculations (Fig. S3). Together, these results extend the individual growth findings and show that cells lacking either the inner or outer membrane transporters are not at a disadvantage when grown in competition with the wild-type strain that encodes both fully intact transporters.

10.1128/mSystems.00116-17.4 FIG S3  Barcoded competitions of individual acquisition mutants and wild-type cells. Download FIG S3, PDF file, 0.4 MB. Copyright © 2017 Costliow and Degnan. 2017 Costliow and Degnan This content is distributed under the terms of the Creative Commons Attribution 4.0 International license.

In contrast, the ΔBioThi mutant was rapidly outcompeted in the absence of thiamine, falling to <0.1% of the population by day 5, despite initially making up 90% of the population. As exogenous thiamine levels were increased, the fitness defect was alleviated. At 10 nM thiamine, the wild type dominated the competition; by day 5, however, the ΔBioThi mutant only dropped to 44% of the population. As the concentrations of thiamine increased to 100 and 10,000 nM, the ΔBioThi mutant showed no fitness defect, maintaining ≥80% of the population throughout the competition (Fig. 4C). Similarly, the ΔΔBioThi-*pnuT* and ΔΔBioThi-*OMthi* strains are dramatically outcompeted in 0 and 10 nM added thiamine to the level that they are not even detectable. As thiamine is increased in concentration, the abundance of the double biosynthesis and transporter mutants stabilizes at ~55% of the population for the ΔΔBioThi-*OMthi* mutant and ~66% of the population for the ΔΔBioThi-*pnuT* mutant, respectively (Fig. 4D and E). The ΔΔTransport and ΔΔΔ mutants were also interrogated for their competitive abilities. We observed severe phenotypes with both mutants in 0, 10, and 100 nM thiamine (Fig. 4F and G). The mutants were rapidly outcompeted by the wild type within the first 3 days and driven below the level of detection by day 5 (Fig. S3). Only in 10,000 nM did the ΔΔTransport mutant and the ΔΔΔ mutant coexist with the wild type—maintaining 40% and 80%, respectively, at day 5. These results essentially mirror the individual growth data for the mutants and suggest that wild-type *B. thetaiotaomicron* does not cooperate with or facilitate the growth of these mutants, indicating that *B. thetaiotaomicron* does not appear to release thiamine to the environment under *in vitro* growth conditions.

### Quantification of cell-associated thiamine.

**FIG 5:**
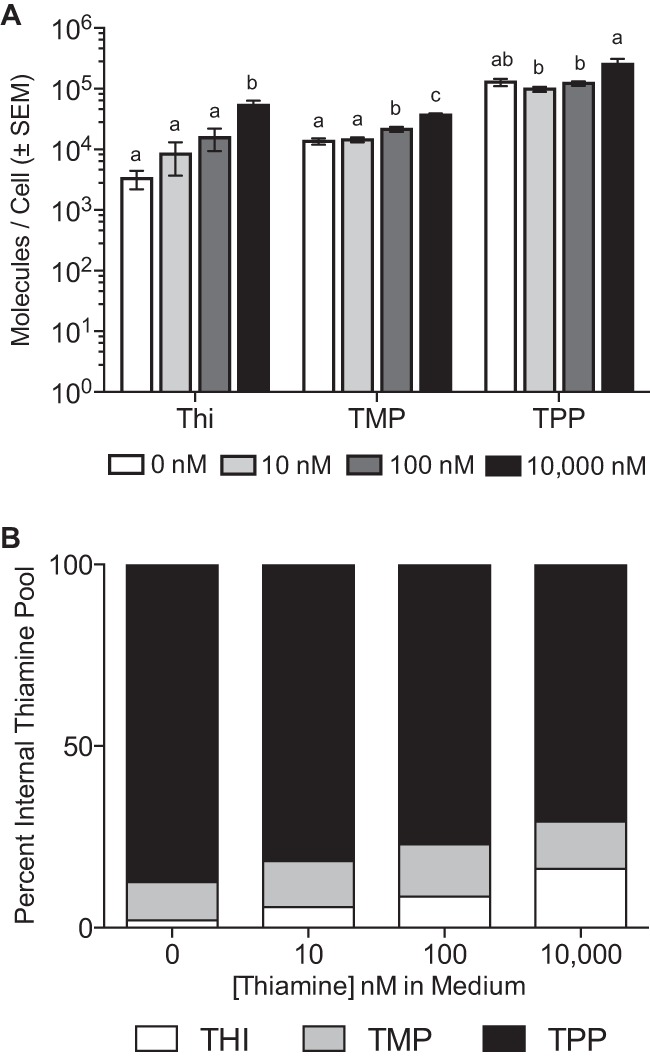
Quantification of thiamine pools in *B. thetaiotaomicron*. (A) Intracellular quantification of thiamine and its phosphorylated moieties indicates that total quantities of thiamine, TMP, and TPP increase to various degrees as exogenous thiamine is increased. The values shown represent the average and standard error of the mean from six biological replicates. Letters for individual moieties indicate groups that are not significantly different from each other (*P* ≤ 0.05, Tukey’s honestly significant difference [HSD] test). (B) The proportions of thiamine and TMP increase as the concentration of exogenous thiamine increases, while the proportion of TPP decreases.

Intracellular concentrations of thiamine, thiamine monophosphate (TMP), and TPP were measured after wild-type *B. thetaiotaomicron* cultures were grown in four different concentrations of thiamine (0, 10, 100, and 10,000 nM). In all the *B. thetaiotaomicron* cultures tested, the cofactor form TPP is the predominant moiety, averaging between 100,000 and 250,000 molecules per cell (Fig. 5A) and constituting 70 to 87% of the total thiamine (Fig. 5B). Therefore, despite a 10,000× increase in the amount of exogenous thiamine, the internal concentration changes no more than 2.5-fold. As such, thiamine moieties appear to be maintained within some homeostatic range. However, as exogenous thiamine levels increase, we did detect marginally significant increases in thiamine and TMP concentrations (Fig. 5A). While TMP maintains 9 to 14% of the thiamine pool in spite of its increased concentrations, thiamine itself increases from ~2 to 16% of the total pool (Fig. 5B). These data suggest thiamine may be entering the cell via nonspecific mechanisms—the same mechanisms responsible for the ability of the ΔΔTransport and ΔΔΔ mutants to grow in 10,000 nM thiamine. As a result, this excess thiamine may impede or exceed the capacity of the thiamine pyrophosphorylase, Tnr3.

### **Identification of thiamine acquisition genes in gut** *Bacteroidetes*.

**FIG 6:**
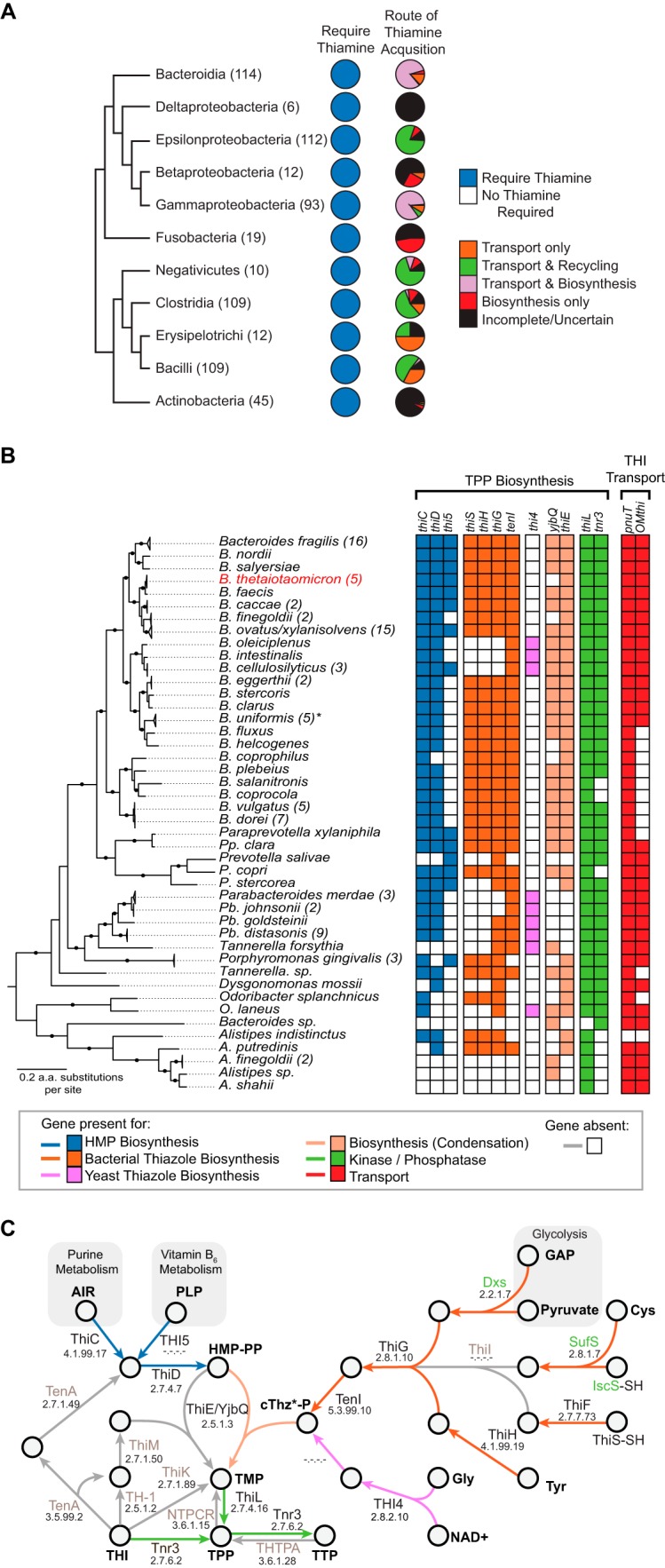
*In silico* predictions of thiamine acquisition pathways. (A) Schematic phylogeny of 11 classes of cultivable gut-associated bacteria and corresponding pie charts indicating the proportion of genomes that encode a thiamine-dependent enzyme and predicted thiamine acquisition strategies. Values in parentheses represent the number of genome sequences in each class. (B) The presence and absence of predicted thiamine biosynthesis and transport genes in diverse gut *Bacteroidetes* are indicated. The phylogeny represents a maximum likelihood reconstruction of gut *Bacteroidetes* species. Numbers in parentheses represent the number of individual strains analyzed, and nodes marked with black circles indicate bootstrap values of ≥75. The asterisk indicates that one of the five *B. uniformis* genomes analyzed is missing *pnuT* and *tnr3*, but this may be an assembly artifact. (C) Schematic representation of thiamine biosynthesis in the *Bacteroidetes*. Arrow colors correspond to the key in panel B. Gene names in gray are missing, and names in green are genes that are not solely dedicated to the biosynthesis of thiamine.

To expand upon the knowledge gained from our experiments in *B. thetaiotaomicron*, we interrogated 641 genomes from 5 phyla and 11 classes of gut bacteria for the presence of thiamine-dependent enzymes. Every one of the 641 genomes encoded at least one thiamine-dependent enzyme (Fig. 6A). We then determined the route by which these bacteria acquire thiamine by examining their genome’s ability to synthesize, transport, or recycle thiamine. Only 48% (307/641) of the genomes investigated have readily identifiable, complete thiamine biosynthesis pathways, implying that many gut microbes likely compete with one another and/or the host for this essential cofactor (Fig. 6A).

All of the gut *Bacteroidetes* genomes examined exhibit evidence of requiring thiamine (*n =* 114). This includes encoding one or more putatively thiamine-dependent enzymes (mean ± SD, 13 ± 2 genes) or a thiamine-binding riboswitch (mean ± SD, 2 ± 1 genes). As such, thiamine biosynthesis and transport genes occur in 94% (107/114) of gut-associated *Bacteroidetes* (Fig. 6B). A minority of sequenced *Bacteroidetes* from the gut encode biosynthesis (*n =* 2) or transport (*n =* 5) alone.

Thiamine biosynthesis occurs in one of two configurations in gut-associated *Bacteroidetes*. The majority of genomes, including *B. thetaiotaomicron*, have a pathway in which 4-amino-2-methyl-5-diphosphomethylpyrimidine (HMP-PP) is synthesized by ThiC and ThiD (blue in Fig. 6B and C) and 2-(2-carboxy-4-methylthiazol-5-yl)ethyl phosphate (cTHz-P) is produced by ThiSFGH-TenI (orange in Fig. 6B and C) (14). These precursors are condensed into thiamine monophosphate by ThiE or YjbQ (tan in Fig. 6B and C), which is then phosphorylated into the biologically active form of thiamine pyrophosphate by ThiL (green in Fig. 6B and C). A handful of gut-associated *Bacteroidetes* have replaced the canonical cTHz-P biosynthetic branch with a THI4-like thiazole synthase enzyme (13/109; EC 2.8.1.10) (fuchsia in Fig. 6B and C). Regardless, all 109 genomes encoding a major biosynthetic operon also encode a TPP riboswitch immediately upstream (18).

None of the gut-associated *Bacteroidetes* possesses a recognizable thiamine ABC transport system similar to the characterized ThiQP-TbpA of Gram-negative bacteria or YkoEDC of Gram-positive bacteria (16, 18). Moreover, *Bacteroidetes* lack the ThiT thiamine transporter and the CytX and ThiW precursor transport proteins (18). Thus, like *B. thetaiotaomicron*, the previously uncharacterized inner and outer membrane transporters (PnuT and OMthi) are widely conserved in gut *Bacteroidetes*, with more than three-quarters (91/114) of the genomes analyzed carrying both *OMthi* and *pnuT* (Fig. 6B). However, heterogeneity in the presence and genomic organization of these genes exists. While *B. thetaiotaomicron* carries a split operon, most genomes contain *OMthi*, *pnuT*, and *tnr3* as a single operon, and others have apparently lost some or all of these genes (e.g., *Bacteroides coprocola*). In most instances, the TPP riboswitch is retained and sometimes duplicated when the operon is split.

## DISCUSSION

Thiamine is an essential cofactor for all living organisms: as such, its availability impacts human health as well as that of our microbiota. Here, we have identified substantial genetic diversity within and among gut bacterial phyla for acquiring thiamine, suggesting diverse strategies are employed leading to opportunities for cooperation and/or competition (Fig. 6A). Among the *Bacteroidetes*, a ubiquitous and abundant gut phylum responsible for carbohydrate fermentation, fatty acid production, and immune function (25, 26), we identified three distinct strategies for thiamine acquisition: biosynthesis and transport, biosynthesis alone, and transport alone. We analyzed the expression profiles, dissected the genetics, and examined the competitive advantages of these strategies in *B. thetaiotaomicron*, demonstrating the importance of both thiamine biosynthesis and transport.

Our results demonstrate that the biosynthesis of thiamine is essential for growth and competition of *B. thetaiotaomicron* in environments with limited thiamine availability. Like many *Bacteroidetes*, *B. thetaiotaomicron* carries a major biosynthetic gene operon (BioThi [*BT0653* to *BT0647*]), which includes essential enzymes for both HMP-PP and cTHz-P production and condensation (Fig. 1A and 6B). Our genome-wide expression profiling identified evidence of thiamine-dependent regulation of this locus that we attribute to the TPP-binding riboswitch preceding this operon. This expression response is consistent with reanalysis of microarray data from *B. thetaiotaomicron* grown both *in vitro* and *in vivo* under thiamine-variable conditions (see Fig. S4 in the supplemental material) (27). Subsequent deletion of the entire biosynthetic operon in *B. thetaiotaomicron* renders it auxotrophic for thiamine, severely impairing growth in media with less than 10 nM thiamine. Both the genetic organization of the locus and a similar growth defect of a *thiC* deletion mutant are seen in *S. enterica* (28). Moreover, the defect in *B. thetaiotaomicron*, which can be complemented by restoring the entire biosynthetic operon in *trans*, is recapitulated during coculture with wild-type cells. Competitions result in the ΔBioThi mutant being outcompeted by the wild-type cells during a multiday coculture experiment under low- or no-thiamine concentrations. We note that thiamine availability in the gut is expected to fluctuate due to various factors, including host genetics, diet, and intestinal adsorption. Measurements of thiamine in the intestinal lumen range between 20 and 2,000 nM (13). As such, a gut microbe’s ability to synthesize its own thiamine is likely most important during transient drops in thiamine availability to enable the microbe’s own growth. We predict that gut microbes that we have identified that are wholly dependent on thiamine transport, such as members of the genus *Alistipes* and many members of the bacilli (Fig. 6), to be adversely affected during such drops in thiamine availability.

10.1128/mSystems.00116-17.5 FIG S4  Microarray measurements of thiamine biosynthesis and transport gene expression. Download FIG S4, PDF file, 0.1 MB. Copyright © 2017 Costliow and Degnan. 2017 Costliow and Degnan This content is distributed under the terms of the Creative Commons Attribution 4.0 International license.

Our global transcriptomic analysis revealed differential responses of thiamine acquisition operons to exogenous thiamine. Under conditions of excess thiamine, we have shown that *B. thetaiotaomicron* downregulates the BioThi operon. However, we found that transcripts for the putative inner (*pnuT*) and outer (*OMthi*) membrane transporter genes adjacent to TPP-binding riboswitches show minimal expression differences (Fig. 1A). These proteins were previously predicted to function in thiamine transport (18), and PnuT was recently shown to transport thiamine in a heterologous host (19). Curiously, individual deletions of these transporters have no effect on the growth of mutants individually or in coculture with wild-type cells. However, deletion of both transport genes in tandem yielded a severe growth defect (Fig. 2). After ruling out an additional role of these transporters for importing a critical precursor (Fig. S1), we speculate that they may play a role in thiamine recycling or that there is an uncharacterized connection between biosynthesis and transport of thiamine. When we measured expression of the BioThi operon in the double transport mutant using RT-qPCR we found expression of *thiC* and *thiS* was downregulated (Fig. 3A and B). This raises the possibility that an unknown protein regulatory mechanism may be dysregulated and repressing biosynthesis in the ΔΔTransport mutant. Regardless, further investigations are warranted to disentangle thiamine biosynthesis and transport and their interconnected pathways.

The dual transport and biosynthesis pathways appear to facilitate the ability of wild-type *B. thetaiotaomicron* to maintain a fairly stable intracellular concentration of the cofactor TPP (<3-fold), despite radical changes in extracellular thiamine concentrations. However, these increases in intracellular concentrations of the thiamine moieties are sufficient to lead to the downregulation of the BioThi operon in addition to a suite of thiamine-dependent pathways, including amino acid metabolism and nucleotide biosynthesis. It is possible that the increased proportion of thiamine to TPP may provide a signal sensed by unknown regulatory mechanisms, outside the regulatory riboswitches that control biosynthesis and transport genes. These additional regulatory factors are likely important for modulating responses to the stress of variable levels of exogenous thiamine.

We observed that competitions between *B. thetaiotaomicron* and thiamine acquisition mutants yielded similar results to growth of the mutants in isolation (Fig. 2 and 4), suggesting that *B. thetaiotaomicron* does not release thiamine into the environment. Confirming this hypothesis, no thiamine was detected in the supernatant of spent cultures of *B. thetaiotaomicron* grown in thiamine-free medium (data not shown). This is not in line with previously published data in which thiamine was still detectable in the feces of a select group of humans who were completely thiamine starved (4). It was inferred that the thiamine detectable in these patients was derived from their microbial communities (4). This leads us to speculate that other gut microbes, including divergent *Bacteroidetes* or *Proteobacteria*, release thiamine as they are the most prevalent thiamine synthesizers. Alternatively, host-driven cell lysis (29), microbe-microbe antagonism and/or intoxication (30), or some gut-specific signal results in the liberation of this essential cofactor.

As an essential small molecule, thiamine appears to have an important role in the persistence of microbes in the gut. The universal requirement for this cofactor and the diverse strategies for acquisition result in scenarios that would force competition or cooperation among gut microbes. Among *B. thetaiotaomicron* mutants encoding a single acquisition strategy, we have observed competitive defects, similar to what has been seen for B_12_ transport (6). However, more complex communities may be crucial to enabling cooperation; thus, modeling the dynamics of thiamine in natural or reconstructed gut communities will be a way forward to understand these dynamics. It thus remains possible that cofactors such as thiamine may be utilized to manipulate gut communities. Ultimately such manipulation may play a role in the combating of diseases such as diabetes, obesity, and cancer.

## MATERIALS AND METHODS

### Bacterial culturing and genetic manipulation.

Routine culturing of *B. thetaiotaomicron* VPI-5482 occurred anaerobically at 37°C in liquid tryptone-yeast extract-glucose (TYG) medium (24) or Difco brain heart infusion (BHI) agar with the addition of 10% defibrinated horse blood (Quad Five, Ryegate, MT). Cultures were grown anaerobically within a vinyl anaerobic chamber with an input gas mixture consisting of 70% nitrogen, 20% carbon dioxide, and 10% hydrogen (Coy Laboratory Products, Grass Lake, MI). *Escherichia coli* S17-1 λ *pir* strains used for cloning and conjugation of suicide vectors were grown in LB medium at 37°C aerobically. Antibiotics were added to the medium when appropriate in the following concentrations: ampicillin, 100 μg/ml; gentamicin, 200 μg/ml; erythromycin, 25 μg/ml; tetracycline, 2 μg/ml; and 5-fluoro-2′-deoxyuridine (FUdR), 200 µg/ml.

Markerless deletions were generated via allelic exchange and confirmed by PCR (6, 31). Briefly, HiFi *Taq* MasterMix (Kapa Biosystems, Wilmington, MA) was used to amplify the right and left flanks of thiamine-associated loci by PCR, which were then combined by splicing by overlap extension (SOE) PCR. Purified SOE products were restriction digested and cloned into pExchange_tdk vector by standard methods (32). Sequence-confirmed vectors were then conjugated from donor *E. coli* S17-1 λ *pir* strains into recipient *B. thetaiotaomicron* strains. Transconjugants were colony purified and screened for successful mutants by PCR (Table 1). Complementation and bar-coded vectors were all constructed in pNBU2 vectors and were introduced into the genome in a single copy (6, 33). Construction and conjugation of pNBU2 vectors was done by the same methods as pExchange_tdk vectors. A complete list of primers and vectors used for this study is provided in Table S3 in the supplemental material.

10.1128/mSystems.00116-17.8 TABLE S3  Primers, vectors, and strains used in this study. Download TABLE S3, XLS file, 0.03 MB. Copyright © 2017 Costliow and Degnan. 2017 Costliow and Degnan This content is distributed under the terms of the Creative Commons Attribution 4.0 International license.

### Thiamine transcriptomic response.

A modified minimal medium was used for all thiamine growth assays (after Varel and Bryant [34] and Scholle et al. [35]). The modified minimal medium consisted of glucose (27.8 mM), ammonium chloride (6.0 mM), l-cysteine freebase (4.1 mM), sodium carbonate (3.8 mM), iron sulfate (0.3 mM), vitamin B_12_ (5.8 μM), a histidine-hematin solution (0.1% 1.9 mM hematin in 0.2 M histidine), 1 M potassium phosphate buffer at a pH of 7.2 (2%), and a premixed mineral solution (5%). The mineral solution consisted of 3 M sodium chloride, 3.6 mM calcium chloride dehydrate, 1.6 mM magnesium sulfate, 1 mM manganese chloride tetrahydrate, and 84.1 μM cobalt chloride hexahydrate. Replicate cultures of wild-type *B. thetaiotaomicron* were grown overnight in 5 ml minimal medium supplemented with 10,000 nM thiamine HCl (THI; >99% pure) (Sigma-Aldrich, St. Louis, MO). Aliquots of each culture were pelleted by centrifugation (1 min at 13,300 × *g*), culture supernatants were decanted, and the cells were washed 4 times in minimal medium with 0 nM thiamine. Cells were inoculated into 10 ml of minimal medium with either 0 or 15 μM thiamine HCl at a final dilution of 1:2,000 in biological duplicate. Cell growth was monitored, and cells were harvested between an OD_600_ of 0.4 and 0.6 on a UV spectrometer. Total RNA was extracted using the Qiagen RNeasy kit and stored at −80°C. Total RNA was DNase treated with DNase I (Thermo Fisher, Waltham, MA) and the NEB DNase treatment protocol. DNase-treated RNAs were recleaned using the Qiagen RNeasy kit (Hilden, Germany), quantitated using a Qubit 2.0 (Life Technologies, Inc., Carlsbad, CA), and stored at −80°C.

RNA was submitted for integrity analysis, rRNA depletion, and library construction at the W. M. Keck Center for Comparative and Functional Genomics at the University of Illinois at Urbana-Champaign.

For targeted gene expression analysis, total RNA was purified as described above and used to generate cDNA libraries using a first-strand cDNA synthesis kit (Thermo Fisher, Waltham, MA). RT-qPCR was performed on a Bio-Rad CFX Connect instrument (Bio-Rad, Hercules, CA) and SYBR Fast MasterMix 2× Universal (Kapa Biosystems, Wilmington, MA) following the manufacturer’s instructions for triplicate biological samples in technical triplicates. Four genes were amplified: the 16S rRNA, *thiC*, *thiS*, and *tnr3* genes. 16S rRNA primers were used as the control gene (6), and novel primers for *thiC*, *thiS*, and *tnr3* were designed using Primer3 (36). Standard curves were used to evaluate the efficiency of the amplification: all 4 genes had *R*^2^ values of ≥0.985 and slopes of between −3.33 and −3.40. Relative expression changes were calculated by the quantification cycle (ΔΔ*C*_*q*_) method (37).

### RNA sequencing analysis.

RNA-seq reads from each sample were quality filtered using a custom R script and fastx_clipper (http://hannonlab.cshl.edu/fastx_toolkit/index.html). Filtered RNA-seq data were analyzed using the Rockhopper program (21) to identify differentially expressed genes between thiamine-replete and thiamine-deficient samples. Reads were aligned to the *B. thetaiotaomicron* VPI-5482 genome, and significantly differentially expressed genes were identified (≥2-fold change and *q* ≤ 0.05).

### Assaying thiamine-dependent growth phenotypes.

*B. thetaiotaomicron* wild-type, mutant, and complemented strains were grown and washed in minimal medium with 0 µM thiamine as described above for RNA-seq. Cells were normalized, diluted to an OD_600_ of 0.0004, and dispensed into 96-well plates with thiamine HCl, thiamine monophosphate HCl (TMP; >95% pure) (Sigma-Aldrich, St. Louis, MO), and thiamine pyrophosphate chloride (TPP; >98%) (Thermo Fisher Scientific, Waltham, MA) at concentrations ranging from 0 to 10,000 nM (modified from Degnan et al. [6]). Cell growth was measured over the course of 25 h using a BioTek Synergy HTX Multi-Mode microplate reader in conjunction with a BioStack3 microplate stacker (BioTek, Winooski, VT). All phenotypic assays were averaged from three to nine biological replicates with three technical replicates each. All phenotypes were calculated as a percentage of WT growth achieved in minimal medium plus 10,000 nM THI at 17.5 h. Pairwise Student’s *t* tests were carried out using GraphPad Prism v6, and *P* values of ≤0.05 were considered significant.

### *In vitro* competition assays.

Cells were grown for 16 h in minimal medium supplemented with 10,000 nM thiamine HCl. Cells were pelleted and washed 4 times with minimal medium. Cell OD_600_ values were normalized to 0.01. Cells were inoculated 1:100 into an Axygen deep-well plate (Corning, Inc., Corning, NY) in biological triplicate for each mutant at 4 different concentrations (0, 10, 100, and 10,000 nM) of thiamine HCl in minimal medium. Cultures were incubated anaerobically at 37°C for 24 h and passaged 1:1,000 into the same medium type they were previously grown in (6, 33). Time points were taken for 4 to 5 days, and samples were saved in medium plus 20% glycerol at −80°C. Samples were prepped for genomic DNA (gDNA) using the HotShot method (38), and the relative abundance of each bar-coded strain was determined using qPCR in reference to a standard curve on a Bio-Rad CFX Connect instrument (Bio-Rad, Hercules, CA) and SYBR Fast MasterMix 2× Universal (Kapa Biosystems, Wilmington, MA) (6). Data were analyzed by the efficiency-corrected Δ*C*_*q*_ method (37).

### Quantification of thiamine in *B. thetaiotaomicron* and medium.

*B. thetaiotaomicron* was grown in either 0, 10, 100, or 10,000 nM thiamine HCl to the mid-log phase (OD_600_ of ~0.5). Cells were pelleted at 10,000 × *g* for 2 min. Cells were washed three times with phosphate-buffered saline (PBS). Cells and medium aliquots were submitted to the Roy J. Carver Biotechnology Centre’s Metabolomics Center at the University of Illinois at Urbana-Champaign for quantification of thiamine, thiamine monophosphate, and thiamine diphosphate. Samples were analyzed with the 5500 QTRAP liquid chromatography-tandem mass spectrometry (LC-MS/MS) system (Sciex, Framingham, MA). Software Analyst 1.6.2 was used for data acquisition and analysis. The 1200 series high-performance liquid chromatography (HPLC) system (Agilent Technologies, Santa Clara, CA) includes a degasser, an autosampler, and a binary pump. The LC separation was performed on a Phenomenex Polar column (4.6 by 100 mm, 4 μm) with mobile phase A (0.1% formic acid in water) and mobile phase B (0.1% formic acid in acetonitrile). The flow rate was 0.4 ml/min. The linear gradient was 0 to 3 min at 95% A, 6 to 9 min at 0% A, and 10 to 15 min at 95% A. The autosampler was set at 10°C. The injection volume was 100 μl. Mass spectra were acquired under positive electrospray ionization (ESI) with an ion spray voltage of +5,000 V. The source temperature was 450°C. The curtain gas, ion source gas 1, and ion source gas 2 were 33, 50, and 65, respectively. Multiple reaction monitoring (MRM) was used for quantitation: thiamine, *m*/*z* 265.0 and 122.0; thiamine monophosphate, *m*/*z* 345.1 and 122.0, and thiamine diphosphate, *m*/*z* 425.1 and 122.0.

### Computational prediction of thiamine-associated genes.

Thiamine biosynthesis and transport genes and thiamine-dependent genes were identified searching for gene-specific TIGRFAM (39) when available or PFAM (40) hidden Markov models with HMMR (--tc_cut) (see Table S4 in the supplemental material) in 641 gut bacteria, including 114 *Bacteroidetes* genomes available in RefSeq from the Human Microbiome Project (see Table S5 in the supplemental material) (41, 42). Predicted annotations were confirmed with BLAST and PFAM hidden Markov models (40, 43). Homologs of *B. thetaiotaomicron* VPI-5482 loci were confirmed and compared among the other 113 *Bacteroidetes* genomes using a reciprocal best BLAST approach. In addition, Infernal 2.0 was used to detect putative TPP riboswitches adjacent to thiamine-associated genes (RF00059) (44, 45). The presence or absence of biosynthetic and transporter genes was used to categorize the route of thiamine acquisition. Microbes capable of complete “biosynthesis” have the ability to synthesize both HMP-PP (*thiC* or *thi5* and *thiD*) and cTHz-P (*thiGH* or *thi4* in addition to *tenI*) and possess thiamine condensation genes (*thiE* and/or *yjbQ*) and a thiamine kinase. Microbes categorized as capable of “recycling” lack the ability to completely synthesize either thiazole and/or HMP-PP *de novo* but retain the thiamine kinase enzymes. Microbes were categorized as capable of “transport” if any gene predicted to transport thiamine was identified. Finally, microbes were categorized as “incomplete/uncertain” if we were unable to identify any thiamine transporters and/or sufficient genes to carry out thiamine biosynthesis or recycling. Additionally, using an approach identical to that described by Degnan et al. (6), a set of 13 core genes conserved among all three domains of life were identified in the 641 gut microbial genomes and aligned, and a phylogeny was reconstructed by the maximum likelihood method.

10.1128/mSystems.00116-17.9 TABLE S4  Search queries used to identify thiamine-dependent and thiamine acquisition genes. Download TABLE S4, XLS file, 0.1 MB. Copyright © 2017 Costliow and Degnan. 2017 Costliow and Degnan This content is distributed under the terms of the Creative Commons Attribution 4.0 International license.

10.1128/mSystems.00116-17.10 TABLE S5  Genomes investigated for thiamine acquisition and thiamine-dependent genes. Download TABLE S5, XLS file, 0.1 MB. Copyright © 2017 Costliow and Degnan. 2017 Costliow and Degnan This content is distributed under the terms of the Creative Commons Attribution 4.0 International license.

### Accession number(s).

RNA-seq data generated for this project have been submitted to the NCBI SRA database under accession no. SRP116143.
