# Supplementary material for: Thiamine Acquisition Strategies Impact Metabolism and Competition in the Gut Microbe Bacteroides thetaiotaomicron
Source: mSystems. 2017 Sep 26;2(5):e00116-17. doi: 10.1128/mSystems.00116-17 (PMC5613172; doi:10.1128/mSystems.00116-17)
Supplement: TEXT S1 [file sys005172138s1.pdf]

SUPPLEMENTAL TEXT: Thiamine acquisition strategies impact metabolism and competition in the gut microbe *Bacteroides thetaiotaomicron*

Zachary A. Costliow[a] and Patrick H. Degnan[a]#

Department of Microbiology, University of Illinois at Urbana-Champaign, Urbana, Illinois, USA[a]

#Address correspondence to Patrick H. Degnan, pdegnan@illinois.edu

Supplemental Results

Characterization of Transport Mutants in Response to Thiamine Precursors

In order to ascertain whether our transport mutant phenotypes were due to a disruption in transport of thiamine precursors instead of thiamine itself. Apart from cysteine, none of the other immediate precursors or intermediates for thiamine biosynthesis are present in minimal medium. Cysteine is both the reducing agent and reduced sulfur source in the medium. Therefore, it is possible that these two genes do not transport thiamine, and instead are required for the acquisition of cysteine as a precursor to enable thiamine biosynthesis. We note that *B. thetaiotaomicron* encodes a putative alanine, serine, and cysteine transporter (*BT1223*) and the biosynthetic pathway for cysteine (*cysE*, *cysK, cysM*). Also, based on the RNAseq results, these cysteine associated genes are expressed in cells grown in minimal medium in the presence or absence of thiamine HCl. However, if *OMthi* and *pnuT* are involved in cysteine transport providing an alternative sulfur source to enable *de novo* biosynthesis of cysteine should reverse the apparent thiamine growth defect. Thus, the cysteine in the medium was replaced with sodium thioglycolate as a reducing agent and sodium sulfide as the sulfur source (S2 Fig) [2]. This change had no effect on the growth of wildtype and the single *OMthi* and *pnuT* mutants indicating that *B. thetaiotaomicron* can use sodium sulfide to biosynthesize cysteine. Moreover, thiamine dependent phenotypes of the double mutants were recapitulated when cysteine was replaced with sodium sulfide as the sulfur source. Together these data indicate that neither of the transporters are involved in cysteine transport.

B. thetaiotaomicron thiamine acquisition mutants were also tested to determine if thiamine precursors or moieties could rescue growth. Similar to the growth assays performed

before thiazole, tyrosine, and GAP were supplemented in the media. Wildtype and the single transport mutants grew normally, exhibiting no growth improvement or impairment in the presence of these metabolites. In addition, in the absence of thiamine none of these metabolites rescued the growth of the double transport mutants at the concentrations tested (0 – 10 μM) (S2 Fig). The failure to rescue the double transport mutant suggests *B. thetaiotaomicron* lacks the ability to import the precursors necessary to biosynthesize thiamine or that some other feedback mechanism exists between biosynthesis and transport.

The response of *B. thetaiotaomicron* mutants to the phosphorylated thiamine moieties were also evaluated for their ability to alter growth phenotypes. *B. thetaiotaomicron* mutants were supplemented with either thiamine monophosphate (TMP) or thiamine pyrophosphate (TPP) and grown as before (S6 Table). Neither TMP, nor TPP rescued growth of the mutants to wildtype levels at a concentration below 1 μM, which is reflected in the EC_{50} values of each mutant increasing on either TMP or TPP. These data suggest that *B. thetaiotaomicron*, like other organisms that rely solely on *pnuT* type transporters like *Acinetobacter baumannii* and *Pseudomonas putida*, cannot transport phosphorylated thiamine moieties [3]. It is likely that the poor growth is due to the presence of dephosphorylated thiamine impurities in commercial TPP and TMP preparations.

RNAseq Results Consistent with Previous Microarray Data

Re-analysis of microarray data exhibit a strong drop in transcript levels of the BioThi operon regulated by a TPP riboswitch *in vitro* when thiamine is replete (TYG vs. MM mean = 13-fold, S5 Fig). This is consistent with either ligand induced transcriptional repression or the result of

transcript instability due to translational regulation. A similar trend is observed with the transport operons, however the baseline expression of the transporters in thiamine depleted conditions is lower than the biosynthetic operon. Moreover, *B. thetaiotaomicron* expression profiles for TPP regulated genes observed in TYG medium are consistent with those from *B. thetaiotaomicron* colonizing the mouse cecum (Cecum vs. MM, mean = 6-fold, S5 Fig) suggesting that thiamine levels are similarly high.

SUPPLEMENTAL METHODS

Re-analysis of Microarray Data

To test for evidence of transcriptional changes in response to thiamine availability we re-analyzed existing microarray data from *B. thetaiotaomicron* growing both *in vitro* and *in vivo* [1]. Briefly, microarrays had been hybridized with total RNA recovered from wildtype *B. thetaiotaomicron* grown to mid-log phase in biological duplicate in minimal medium with no thiamine added (8h), in TYG which has excess thiamine (3.5h) or from the ceca of two monoassociated gnotobiotic NMRI mice (GSE2231) [1]. Raw GeneChip data were normalized and model-based expression values (PM-MM model) were compared using DNA-Chip Analyzer (v2011.05) as described [1]. Mean and standard deviations of the normalized expression values were calculated and significantly differentially regulated genes were detected with *t*-test, $p < 0.05$ using FDR correction.

References

1. Sonnenburg JL, Xu J, Leip DD, Chen C-H, Westover BP, Weatherford J, Buhler JG, Gordon JI. Glycan foraging *in vivo* by an intestine-adapted bacterial symbiont. Science. 2005;307: 1955–1959. doi:10.1126/science.1109051
2. Varel VH, Bryant MP. Nutritional features of *Bacteroides fragilis* subsp. fragilis. Appl Microbiol. 1974;28: 251–257.
3. Genee HJ, Bali AP, Petersen SD, Siedler S, Bonde MT, Gronenberg LS, Kristensen M, Harrison SJ, Sommer MO. Functional mining of transporters using synthetic selections. Nat Chem Biol. 2016;12: 1015–1022. doi:10.1038/nchembio.2189

Supplemental Tables

Table S1. Samples used for RNA sequencing experiment.

Table S2. Effective concentrations for 50% WT growth represented in nM of thiamine required.

Table S3. Primers, vectors, and strains used in this study.

Table S4. Search queries used to identify thiamine-dependent and thiamine acquisition genes.

Table S5. Genomes investigated for thiamine acquisition and dependent genes.

Supplemental Figure Legends

FIG S1. Putative *B. thetaiotaomicron* thiamine transporters are specific for thiamine

The specificity of the thiamine dependent phenotypes of the thiamine acquisition mutants were tested using media supplemented with thiamine precursors. To disentangle cysteine's role as a sulfur source and reducing agent, growth analyses were carried out with medium

supplemented (A) cysteine and thioglycolate or (B) Na_2S and thioglycolate. Other commercially available thiamine precursors (C) Tyrosine, (D) GAP, and (E) Thiazole were also analyzed to determine if they could rescue thiamine growth phenotypes. In all panels thiamine was either at (+) 10,000 nM or (-) 0 nM in the and experiments were performed in biological duplicate.

FIG S2. Growth phenotype of ΔΔTransport double transport Complement

ΔΔTransport mutant was complemented with both the inner (*pnuT*) and the outer membrane transporter (*OMthi*) under the natural promoter of *pnuT* and expressed at a non-native locus in the ΔΔTransport mutant. The double complement allows for a marked improvement in growth at low nanomolar concentrations of thiamine and partially relieves the fitness defect that we observe in the ΔΔTransport mutant.

FIG S3. Barcoded competitions of individual acquisition mutants and wildtype cells

Abundance of wildtype (WT) and thiamine acquisition mutants during multiday competitions were tracked using qPCR. Wildtype *B. thetaiotaomicron* was competed against (A) ΔBioThi, (B) Δ*pnuT*, (C) Δ*OMthi*, (D) ΔΔTransport, (E) ΔΔΔ mutants, (F) ΔΔBioThi-*OMthi*, and (G) ΔΔBioThi-*pnuT* in 0 nM, 10 nM, 100 nM, and 10,000 nM thiamine. All competitions were performed in biological triplicate and error bars represent standard error of the mean.

FIG S4. Microarray data of thiamine biosynthesis and transport genes

Expression of *B. thetaiotaomicron* genes involved in thiamine biosynthesis and transport during growth with known or assumed thiamine availability. Asterisks and brackets indicated

significant expression differences between samples (FDR corrected *t*-test, $p < 0.05$) and shaded areas indicate genes encoded in the same operon.
