# Supplementary material for: Thiamine Acquisition Strategies Impact Metabolism and Competition in the Gut Microbe Bacteroides thetaiotaomicron
Source: mSystems. 2017 Sep 26;2(5):e00116-17. doi: 10.1128/mSystems.00116-17 (PMC5613172; doi:10.1128/mSystems.00116-17)
Supplement: FIG S1 [file sys005172138sf7.pdf]

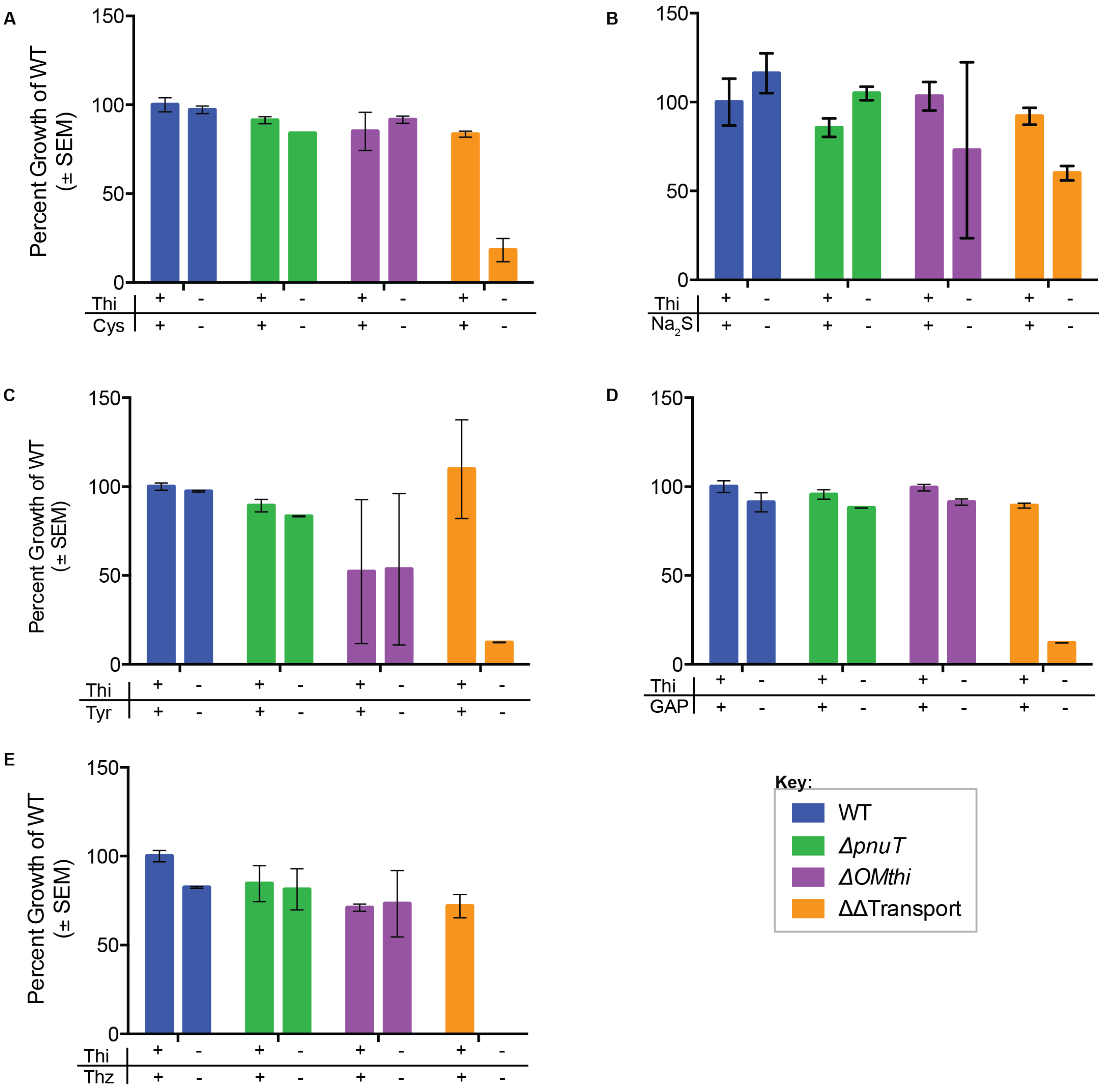

FIG S1

FIG S1. Putative *B. thetaiotaomicron* thiamine transporters are specific for thiamine

The specificity of the thiamine dependent phenotypes of the thiamine acquisition mutants were tested using media supplemented with thiamine precursors. To disentangle cysteine's role as a sulfur source and reducing agent, growth analyses were carried out with medium supplemented (A) cysteine and thioglycolate or (B) Na_2S and thioglycolate. Other commercially available thiamine precursors (C) Tyrosine, (D) GAP, and (E) Thiazole were also analyzed to determine if they could rescue thiamine growth phenotypes. In all panels thiamine was either at (+) 10,000 nM or (-) 0 nM in the and experiments were performed in biological duplicate.
