# Supplementary material for: Thiamine Acquisition Strategies Impact Metabolism and Competition in the Gut Microbe Bacteroides thetaiotaomicron
Source: mSystems. 2017 Sep 26;2(5):e00116-17. doi: 10.1128/mSystems.00116-17 (PMC5613172; doi:10.1128/mSystems.00116-17)
Supplement: FIG S2 [file sys005172138sf8.pdf]

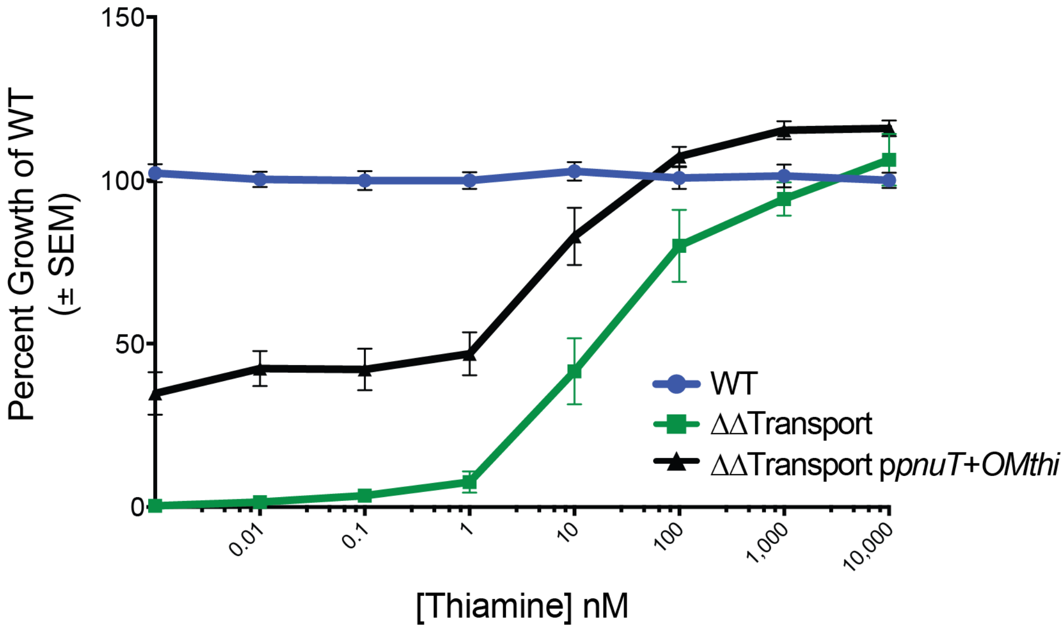

FIG S2

FIG S2. Growth of dually complemented ΔΔTransport mutant.

ΔΔTransport mutant was complemented with both the inner (*pnuT*) and the outer membrane transporter (*OMthi*) under the natural promoter of *pnuT* and expressed at a non-native locus in the ΔΔTransport mutant. The double complement allows for a marked improvement in growth at low nanomolar concentrations of thiamine and partially relieves the fitness defect that we observe in the ΔΔTransport mutant.
