# Supplementary material for: Thiamine Acquisition Strategies Impact Metabolism and Competition in the Gut Microbe Bacteroides thetaiotaomicron
Source: mSystems. 2017 Sep 26;2(5):e00116-17. doi: 10.1128/mSystems.00116-17 (PMC5613172; doi:10.1128/mSystems.00116-17)
Supplement: FIG S3 [file sys005172138sf9.pdf]

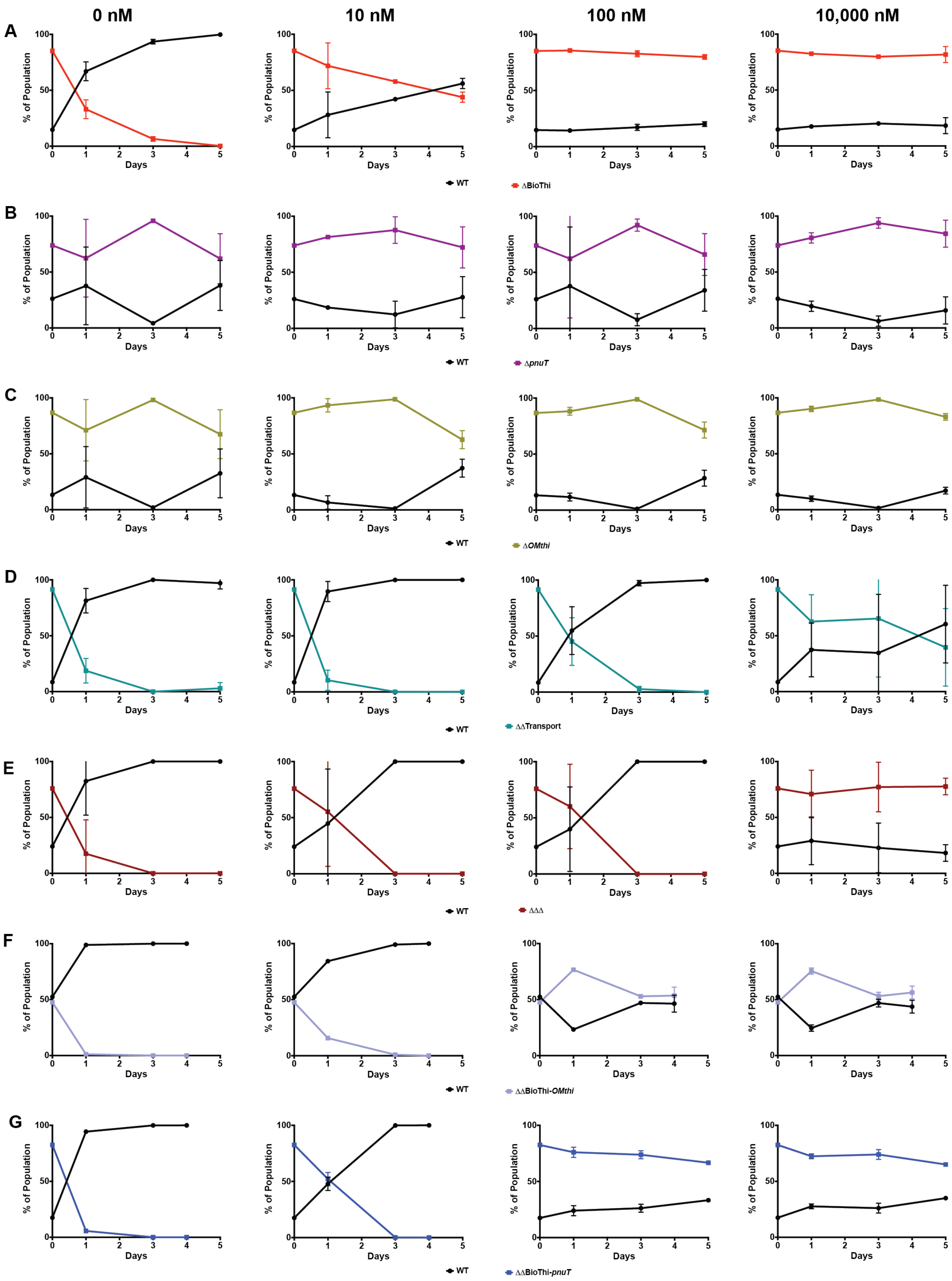
0 nM
10 nM
100 nM
10,000 nM
A
B
C
D
E
F
G
% of Population
Days
WT
ΔBioThi
Δ*pnuT*
Δ*OMthi*
ΔΔTransport
ΔΔΔ
ΔΔBioThi-*OMthi*
ΔΔBioThi-*pnuT*

FIG S3. Barcoded competitions of individual acquisition mutants and wildtype cells

Abundance of wildtype (WT) and thiamine acquisition mutants during multiday competitions were tracked using qPCR. Wildtype *B. thetaiotaomicron* was competed against (A) ΔBioThi, (B) Δ*pnuT*, (C) Δ*OMthi*, (D) ΔΔTransport, (E) ΔΔΔ mutants, (F) ΔΔBioThi-*OMthi*, and (G) ΔΔBioThi-*pnuT* in 0 nM, 10 nM, 100 nM, and 10,000 nM thiamine. All competitions were performed in biological triplicate and error bars represent standard error of the mean.
