# Supplementary material for: Thiamine Acquisition Strategies Impact Metabolism and Competition in the Gut Microbe Bacteroides thetaiotaomicron
Source: mSystems. 2017 Sep 26;2(5):e00116-17. doi: 10.1128/mSystems.00116-17 (PMC5613172; doi:10.1128/mSystems.00116-17)
Supplement: FIG S4 [file sys005172138sf10.pdf]

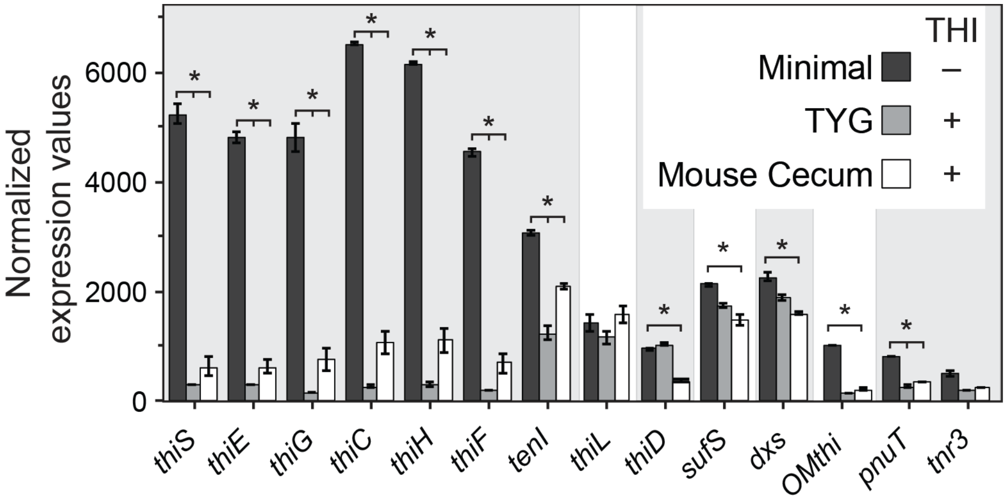

FIG S4

FIG S4. Microarray measurements of thiamine biosynthesis and transport gene expression. Expression of *B. thetaiotaomicron* genes involved in thiamine biosynthesis and transport during growth with known or assumed thiamine availability. Asterisks and brackets indicated significant expression differences between samples (FDR corrected *t*-test, $p < 0.05$) and shaded areas indicate genes encoded in the same operon.
